# Supplementary material for: Macrophage-Derived Factors with the Potential to Contribute to the Pathogenicity of HIV-1 and HIV-2: Roles of M-CSF and CXCL7
Source: Int J Mol Sci. 2025 May 23;26(11):5028. doi: 10.3390/ijms26115028 (PMC12155501; doi:10.3390/ijms26115028)
Supplement: Supplementary file 1 [file ijms-26-05028-s001.zip › ijms-3585343-supplementary.pdf]

Macrophage-derived factors with the potential to contribute to pathogenicity of HIV-1 and HIV-2: Role of M-CSF and CXCL7

Chunling Gao[1^], Joseph Kutza[1], Ouyang Weiming[2^†], Tobias A. Grimm[1], Karen Fields[1*], Carla S.R. Lankford[1], Franziska Schwartzkopff[1], Mark Paciga[1], Ana Machuca[3], Linda Tiffany[1], Tzanko Stantchev[1†] and Kathleen A. Clouse[1]

[1] Division of Pharmaceutical Quality Research Four (DPQR-IV), Office of Pharmaceutical Quality Research, Center for Drug Evaluation and Research, U. S. Food and Drug Administration, 10903 New Hampshire Avenue, Silver Spring, MD 20993
[2] Division of Pharmaceutical Quality Research Three (DPQR-III), Office of Pharmaceutical Quality Research, Center for Drug Evaluation and Research, U. S. Food and Drug Administration, 10903 New Hampshire Avenue, Silver Spring, MD 20993
[3] Laboratory of Molecular Virology, Division of Emerging and Transfusion Transmitted Diseases (DETTD), Center for Biologics Evaluation and Research (CBER), U. S. Food and Drug Administration, 10903 New Hampshire Avenue, Silver Spring, MD 20993
[^] Contributed equally to this work.
[*] Deceased
† Address correspondence and reprint requests to:
Dr. Weiming Ouyang, Phone: (240) 402-7300; E-mail: weiming.ouyang@fda.hhs.gov
Dr. Tzanko Stantchev, Phone: (240) 402-7400; E-mail: Tzanko.Stantchev@fda.hhs.gov

Supplementary Material

Figure S1. M-CSF induction in MDM following parallel infection with HIV-1 or HIV-2 strains. Supernatants were harvested from MDM that were uninfected or infected in parallel with individual isolates of HIV-2 B4, B5, B7, B8, Rod or HIV-1 92UG024, BCF03, and Ada at the peak time of viral replication. The levels of M-CSF present in the supernatants were measured by M-CSF-dependent proliferation of M-NFS-60 cells. The concentrations of M-CSF in the supernatant of each infection group were normalized to the expression levels of M-CSF in the supernatants harvested from uninfected MDM and presented as relative M-CSF levels. Each symbol represents one individual donor. Data shown were statistically analyzed using the One-Way ANOVA (n = 2 - 3). The multiple testing corrections were performed by the Dunnett's test using HIV-1 Ada as the control group for multiple comparisons.

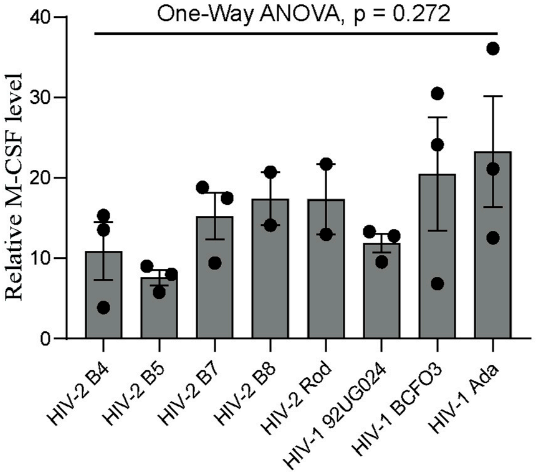

Figure S2. CXCL7 expression in MDM following parallel infection with HIV-1 or HIV-2 strains. Supernatants were harvested from MDM that were uninfected or infected in parallel with individual isolates of HIV-2 B4, B5, B7, B8, Rod or HIV-1 92UG024, BCF03, and Ada at the peak time of viral replication. The levels of CXCL7 in the supernatants were determined by ELISA. The concentrations of CXCL7 in the supernatant of each infection group were normalized to the expression levels of CXCL7 in the supernatants harvested from uninfected MDM and presented as relative CXCL7 levels. Each symbol represents an individual donor. Data were shown as the mean ± SEM (n = 2 - 3) and the asterisks on the long bar line depicts a significant difference among the 8 HIV-1 and HIV-2 infection groups, which were analyzed by the One-Way ANOVA. The asterisks on the short bar lines depict significant differences (*, $p<0.05$ and **, $p<0.005$) between the HIV infection groups under the bar lines and the control HIV-1 Ada infection group that were assessed by multiple comparisons using the Dunnett's test.

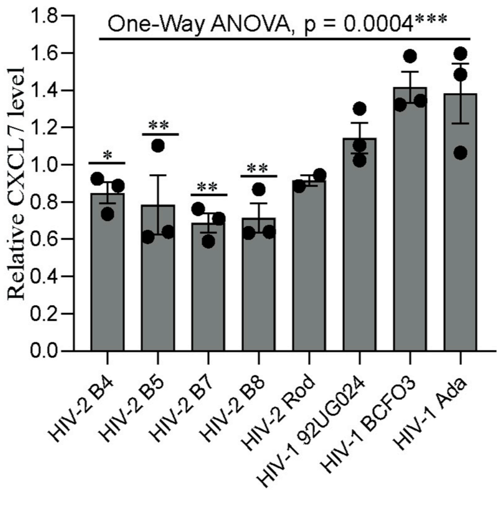

Figure S3. The role of CXCL7 in the supernatants harvested from HIV-infected MDM in chemoattracting lymphocytes. Human MDM were uninfected (Cell only) or infected with the indicated individual HIV isolates. Supernatants were harvested at the peak time of virus replication. The chemotaxis activities of the harvested supernatants were assessed using the 96-well cell migration assay kit (Catalog# 3465-096-K) purchased from Trevigen Inc (Gaithersburg, MD) following the manufacturer's instructions. Briefly, 50 thousand elutriated fresh human lymphocytes were added to the top chambers of transwell plates, which were incubated in bottom chambers containing 150µL harvested supernatant with or without anti-CXCL7 antibody (0.1µg/mL) (Catalog# MAB393, R&D Systems, Minneapolis) at 37ºC for 24 hours. Following the incubation, migrated cells were determined by incubating with 100µL of Cell Dissociation Solution/Calcein-AM at 37ºC for one hour and measuring fluorescence (485 nm excitation, 520 nm emission). Relative fluorescence units depict the number of migrated cells. Data shown were results of supernatants harvested from MDM prepared from two different donors (n = 2). Data from the four groups without anti-CXCL7 antibody (black bars) were analyzed using the One-Way ANOVA with the Dunnett's test for multiple testing corrections that use HIV-1 Ada as the control group. The asterisk on the black bar depicts a significant difference (p<0.05) between the HIV-2 B4 and HIV-1 Ada infection groups. The asterisks on the gray bars depict significant differences (p<0.05) between samples analyzed with or without anti-CXCL7 antibody.

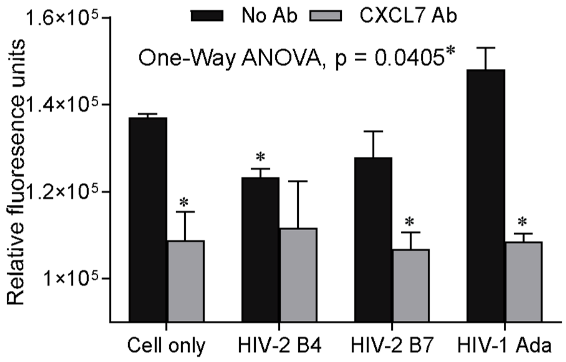

Table S1. Virus replication and M-CSF induction in MDM infected with HIV-1 isolates

Donor #7	HIV-1 92UG024		HIV-1BCF03		HIV-1 Ada	
	RT (fold) [1]	M-CSF (fold) [1]	RT (fold)	M-CSF (fold)	RT (fold)	M-CSF (fold)
Day 3	17.01	1.27	42.01	-0.21	0.52	0.37
Day 6	30.29	0.51	114.18	2.25	3.42	0.39
Day 9	27.36	2.35	113.83	3.44	9.72	2.48
Day 12	28.01	3.62	149.44	3.95	34.74	3.85
Day 15	29.31	9.02	106.42	6.87	54.81	12.56
Day 18	23.37	9.56	61.83	2.48	56.99	8.37
Day 21	19.41	2.57	44.59	0.75	51.71	3.43
Day 24	9.78	5.48	20.12	2.17	27.62	4.77
Day 27	8.32	4.27	13.35	0.95	19.83	4.94
Day 30	6.49	2.07	10.39	-0.56	19.28	1.21
Day 33	7.74	1.72	15.27	-0.39	20.15	1.64
Day 36	7.60	2.20	10.76	0.10	21.56	1.91
Day 39	5.49	2.75	9.35	-0.09	16.13	3.21

[1] Fold change was calculated using the formula: (RT/M-CSF of HIV-1-infected MDM – RT/M-CSF of uninfected MDM) ÷ (RT/M-CSF of uninfected MDM)
